# The “Social Brain,” Reciprocity, and Social Network Segregation along Ethnic Boundaries

**DOI:** 10.1007/s12110-020-09382-5

**Published:** 2021-01-11

**Authors:** Michael Windzio

**Affiliations:** University of Bremen, SOCIUM, PO Box 330 440, 28334 Bremen, Germany

**Keywords:** Cognitive modes, Social networks, Stochastic actor-based models, Ethnic boundaries, Reciprocity, Rationality, Social brain

## Abstract

How does segregation along ethnic boundaries emerge in social networks? Human evolution resulted in highly social beings, capable of prosociality, mindreading, and self-control, which are important aspects of the “social brain.” Our neurophysiologically “wired” social cognition implies different cognitive *goal frames*. In line with recent developments in behavioral theory, the present study defines network ties as *episodes* of social exchange. This dynamic definition can account for shifts in goal frames during an exchange episode: whereas deliberate choice and *hedonic* or *gain* goals drive the initiation of a tie, given the opportunity structure, the *normative goal frame* activates a strong dynamic effect of *reciprocity*, which limits actors’ choice set and appears as “self-organization” at the network level. Longitudinal analyses of 18 birthday party networks comprising 501 students support the definition of network ties as exchange episodes, as well as the relevance of humans’ inherent tendency to reciprocate. However, reciprocation is much stronger in dyads of the same ethnicity than in dyads of different ethnicities. Network segregation along ethnic boundaries results from deliberate decisions during the initiation of an episode, but also from different commitments to reciprocity during the ongoing exchange process, depending on intra or interethnic dyadic constellations.

The neurophysiological disposition to reciprocate is a product of human evolution and plays a crucial role in the emergence of social networks. Network ties emerge and exist in the form of *episodes of social exchange*, beginning with the initiation of an exchange, its progression over time, and its end. According to *goal-framing theory* (Lindenberg 2015b), a goal frame limits the scope of social cognition and decision-making. When reciprocating, the *normative* goal frame usually strives for the cognitive foreground and pushes other goals into the background.

Based on the goal-framing perspective, which is a refinement of Dunbar’s (2003) “social brain” hypothesis, the present study analyzes the effect of reciprocity on the evolution of social exchange networks. Results of longitudinal analyses of network evolution show that the normative goal frame, and the resulting commitment to reciprocity, is highly important during an ongoing social exchange episode. However, the inclination to reciprocate is considerably lower in dyads of different ethnic background. Network segregation along ethnic boundaries emerges not only from opportunities to initiate a network tie (e.g., from residential segregation) but also from the ongoing social exchange process and varying commitments to reciprocity.

It will be argued below in the “Theory and Research” section that goal-framing theory is superior to “classic” rational choice theory when explaining the emergence of network ties and the ethnic segregation of networks. Goal-framing theory describes human social cognition in a more realistic way: “Rational choice reconstructions of solidarity … more or less ignore the architecture of the social brain and thereby also cannot deal with the non-strategic aspects of cooperative behavior” (Lindenberg 2015c:32). Our understanding of networks, social cohesion, and ethnic boundaries remains incomplete if we ignore the neurophysiological architecture of the social brain and its evolution.

Multilevel selection during human evolution enhanced the ability to cooperate, but also to distinguish between in- and out-group. According to the “social brain” thesis (Dunbar 2003; Gamble et al. 2014), human sociality became physically embodied by the coevolution of brain size, particularly the neocortex, and the size of cohesive groups. Cognitive capacities to recognize others’ intentions gained in importance in increasingly complex social settings. Evolution favored the “intentional stance” (Dennett 2017:93), the life-saving assumption of being surrounded by subjects who might intend, for example, to eat, to escape, or to mate. In conjunction with pair bonding and “cooperative breeding,” this further developed into the capacity to self-regulate, to think about others’ mental states, and to apply complex “theories of mind” (Hrdy 2009; Lindenberg 2015c). Acting in such a way as members of larger groups is a distinctive feature of humans.

While the neurophysiological foundation of social cognition and morality is now taken for granted in evolutionary anthropology and psychology (Boehm 2012; Greene 2015; Haidt 2012), sociology could also improve some of its core concepts, such as cooperation, reciprocity, and social cohesion, by systematically considering the intrapersonal dynamics of cognitive modes (Turner 2021).

Assuming different cognitive modes is in line with the “social brain” thesis, with recent developments in the neuroscience of social decision-making (Rilling et al. 2002; Rilling and Sanfey 2011), and also with the assumption of a “natural” disposition to reciprocate (Bowles and Gintis 2011; Diekmann 2004; Greene 2015). This disposition appears as “self-organization” at the aggregate level of the social network: network ties occur because other ties already exist.

The present paper elaborates the following argument: social interaction in modern, diverse mass societies requires a specific capacity of self-regulation to calibrate different goal frames, and to regulate the interplay between normative, hedonic, and gain goal frames in a manner appropriate to a given situation. Cohesion of social networks requires reciprocity resulting from a strong normative goal frame which, however, is comparatively weak in social exchange between different ethnic groups.

Using data on school-class networks of children’s and adolescents’ birthday parties (Windzio 2012), empirical results of stochastic actor-oriented models (SAOMs) for the evolution of networks (Snijders, van Bunt, and Steglich 2010) support the hypothesis that the reciprocity effect is considerably smaller in dyads of different ethnicities.

## Theory and Research

**Fig. 1:**
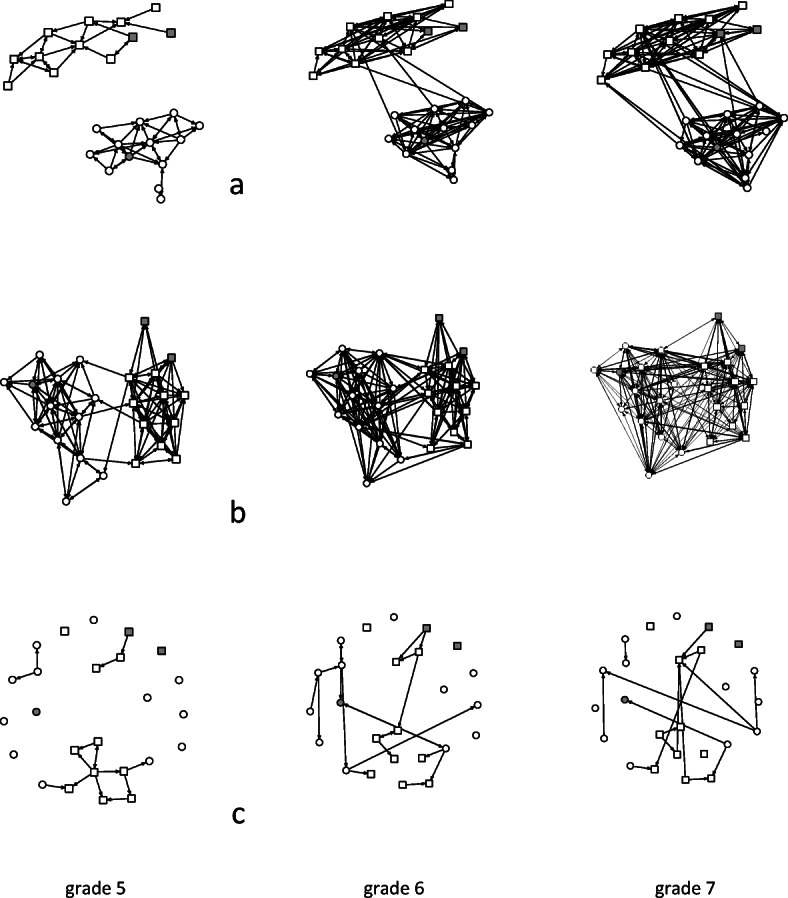
Evolution of networks over three measurement occasions: (**a**) birthday, (**b**) friendship, (**c**) contact among parents

By inviting peers to a birthday party or by accepting an invitation to one, children’s and adolescents’ friendships are publicly confirmed, so that birthday parties become important for a child’s position in the prestige hierarchy of the peer-group. From the parent’s point of view, birthday parties are an exchange of goods and children between families and households (Clarke 2007; Hochschild 2005; Windzio 2012), where norms of reciprocity are particularly strong. Figure 1 shows the evolution of a network in three different dimensions in the fifth, sixth, and seventh school grades. The network generator of the outcome variable is the following: “Whose birthday party did you attend?”

Gray indicates pupils from migrant families; white, from nonmigrant families. Circles indicate girls; squares, boys. How can we explain the dynamics of the birthday party network?

### “Self-Organization” in Social Networks

The concept of “network self-organization” (Robins 2015:34) became prominent in the 1970s, when neurobiologists were looking for a theory to explain the reestablishment of network topologies in damaged neural systems. A purely genetic theory of regeneration due to cell-by-cell genetic determination would require an unrealistic amount of genetic information. The reestablishment of the neural network can be modeled much more simply by mutual reinforcement of cellular activity in neighborhoods. Assuming basic axonal activities in a simulation model, such as continually putting out branchlets which become withdrawn if not reinforced, and genetic information limited only to a small set of “polarity marker-cells” as landmarks in the topological map, sufficiently explains the reemergence of the structure (Willshaw and von der Malsburg 1976).

In social networks, persons decide whether they will establish, maintain, or dissolve ties. Nevertheless, “network self-organization” is more than just a metaphor for the evolution of networks. The concept draws our attention to *constraints on social decision-making*. The way we interact in everyday life and react to other people is anything but arbitrary. It is highly structured by our institutionalized environment, but also influenced by our biology. After an exchange relationship has been initiated and accepted, reciprocating is often regarded as a taken-for-granted matter, so the choice-set becomes strongly limited (Meyer 2010). From a macro perspective, the network appears to an observer as an evolving social system, in which existing ties stimulate the emergence of new ties. For instance, “social capital” is the realistic expectation of getting support from the social network. Social capital is not exhausted by use, but can even be enhanced when expectations of reciprocity emerge (e.g., in a working group, friendship, school, or neighborhood; Coleman 1990:321). The basic idea of network self-organization is similar: the presence of ties in a network increases either the stability of other existing ties or the probability of new ties—for example, by *transitive closure* (“friends of my friends are my friends”) or *reciprocity* (“tit-for-tat”) (Robins 2015:34). Although network research is commonplace in the social sciences today, it lacks a systematic foundation in a theory of human action and behavior which combines arguments on appropriate action in a given situation, or “rational choice,” on the one hand, and network self-organization. on the other. Most real-life networks result from opportunities and choice, but also from taken-for-granted routines and emotions, which appear as “self-organization” at the network level when actors strongly tend toward reciprocity.

This argument will be elaborated in the following section, where three propositions related to the microfoundation of network ties will be made. First, instead of misconceiving network ties as stable entities, the focus should be on their dynamics (Kuwabara and Sheldon 2012). Network ties are *episodes of social exchange*. Second, cooperation and reciprocity have a neurophysiological basis in the “social brain.” Third, when applied to network ties, behavioral theory should distinguish between different *goal frames* (Lindenberg 2008), activated by situational cues.

### Network Ties as Episodes of Social Exchange

Sociological network researchers in the 1970s, particularly those at Harvard, developed a variety of global and local network measures (Prell 2012:42), but most of them were cross-sectional. The flipside of the Harvard Structuralists’ great success was the reification of the network as a given social structure: “Sociological research has focused extensively on networks in stasis, paying far less attention to how individual exchange relations emerge, evolve, stabilize or vanish over time” (Kuwabara and Sheldon 2012:271). Cross-sectional manifestations of networks are only snapshots of dynamic sequences of decisions on tie creation, maintenance, or dissolution at the micro level (Snijders 1996). Research on social exchange, in contrast, analyzes the *dynamics* in “episodes of reciprocal exchange” (Molm et al. 2012:161). In working groups, exchange often begins with rational reasoning and instrumental motives, but during recurrent interactions over time, positive emotions gain in importance (Lawler et al. 2015).

Kuwabara and Sheldon (2012) study different dynamics of social exchange, which they call “testing the water” and “leap of faith.” The former is a tentative, incremental development of social commitment in a situation of uncertainty and perceived risks, whereas the latter indicates a nearly immediate onset of frequent “high-stakes interactions.”

If interethnic network ties tend to develop more in terms of “testing the water” and have a lower likelihood of reciprocity, ethnic segregation will also result from the exchange process itself, and not just from preferences when initiating a tie, given the respective opportunity structure. The following sections further elaborate why cognitive modes becoming active during the exchange episode can further increase ethnic network segregation.

### The Social Brain and Reciprocity

Evolutionary anthropology and psychology agree that reciprocity, cooperation and investment in collective goods pay off for the group. Between-group competition or conflict has been an evolutionary driving force to solve the free rider problem: cooperative groups had an advantage over noncooperative groups but also had to defend their resources against intruders from the outside. This led to *parochial altruism*, a form of solidarity in which actors are well aware of group boundaries and favor the in-group (Bowles and Gintis 2011) or even show negative feelings toward the out-group (Sapolsky 2018:400).

Studies in the neurosciences support the notion of a strong tendency toward reciprocity and cooperation. In a series of experiments, Sakaiya et al. (2013) showed in an iterative prisoner’s dilemma study that others’ nonreciprocity correlated with activation in the amygdala (which mostly generates negative feelings, such as fear or disgust; Sakaiya et al. 2013:8). Nonreciprocal partners stimulate negative feelings, whereas reciprocal partners stimulate positive ones, which is reflected in correlated brain-area activation. Experiments on neural activities in social interaction highlight how specific brain regions are coordinated when persons reciprocate trust (van den Bos et al. 2009). Indirect “pay-it-forward” reciprocity corresponds with neurologically measurable emotional rewards (Watanabe et al. 2014). Actors who break a given promise to reciprocate trust show higher activity in brain regions that are usually involved in cognitive conflict and control. Our inherited disposition to reciprocate is even strong enough to become exploited for manipulative purposes (e.g., for commercial marketing or other kinds of social influence; Cialdini 2007: chap. 2). Violating expectations of reciprocity is a psychological challenge, as J. Rilling and A. Sanfey conclude in their overview: “whether through innate, genetic predispositions or through socialization, the tendency to reciprocate altruism appears to become ingrained in our biology and overridden only with cognitive effort” (Rilling and Sanfey 2011:30).

Reciprocal altruism became part of human nature (Turner 2021) during the autocatalytic (i.e., self-reinforcing) take-off period of gene-culture coevolution (Henrich 2016:314), when group and brain size suddenly increased and social interaction became more complex (Gamble et al. 2014), as proponents of the *cumulative cultural brain* hypothesis (Muthukrishna et al. 2018) argue. As a result, cognitive states change when persons process new information while deciding either on initiating a network tie or on reciprocating. In addition, the micro-level effect of reciprocity in a network’s process of self-organization might be moderated by the salience of objects (Kuwabara and Sheldon 2012:258), in particular by the ethnic origin of other persons involved in the exchange.

### Reciprocity in Goal-Framing Theory and Ethnic Boundaries

Although fast vs. slow cognition (Esser and Kroneberg 2015; Kahneman 2012) might play a role in social tie creation (e.g., in romantic relationships), there usually is some time during an exchange episode to reflect on the relationship and on reciprocity. Lindenberg’s goal-framing theory is a more appropriate approach to explain how our social brains are involved in social networks and in the reproduction of ethnic boundaries: because of the advantage of collective goods at different levels of aggregation, humans developed *social rationality* or *group-mindedness* (Lindenberg 2015a). Since the working memory’s capacity increased during human evolution (Gamble et al. 2014), humans became increasingly able to keep track of who initiated which actions, to develop a “theory of mind,” and to cognitively master higher-order intentionality: a person can assume that another person thinks about a third person’s intention to betray an incautious fourth person (Dennett 2017:288; Gamble et al. 2014:52). In addition, social order became dependent on individuals’ capacity of self-regulation (Lindenberg 2013), which is either a result of multilevel selection or of ostracism of uncooperative individuals, free riders, or “bullies” from the community. According to the former argument, selection operated at the individual *and* at the group level, but potentially at opposite directions: whereas uncooperative cheaters might increase their individual fitness within the community, communities with many cheaters fail to provide collective goods and are therefore outcompeted at the between-group level—by conflict or simply by niche competition (Turchin 2016:84). According to the ostracism thesis, self-control resulted from group punishment against bullies and free riders. Ostracism and capital punishment severely reduced the fitness of deviants and altered the gene pool (Boehm 2012:149): “we may assume that thieves, cheaters, and, especially, alphas were not going away quietly; that many were killed or otherwise disadvantaged along their way; and that the human capacity for self-control was advancing as a result of all this drastic *social selection*” (Boehm 2012:163).

One outcome of these selective processes is the highly social nature of humans. Being concerned about personal reputation became a crucial aspect of our moral consciousness, so that moral emotions (e.g., feelings of shame and guilt) and the capacity of self-regulation seem to be human universals (Boehm 2012:20; Lindenberg 2015c). Both selective mechanisms stabilized the human capacity to calibrate a complex set of overarching goals, also called “mindsets” (Lindenberg 2015b). Once active and in the cognitive foreground, overarching goals organize the *framing* of the situation. Goals frame situations and stimulate specific cognitions and evaluations (Lindenberg 2013:82).

**Fig. 2:**
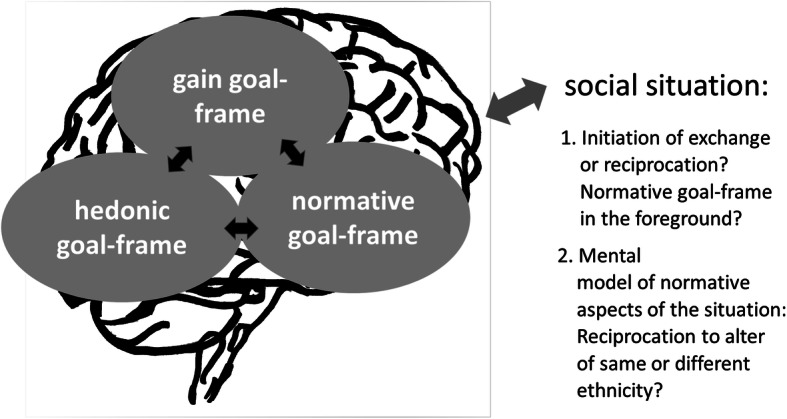
The “social brain” and network segregation along ethnic boundaries in episodes of social exchange

Goal-framing theory considers three “master goal frames” (Lindenberg 2008). First of all, when the *hedonic* goal frame is in the cognitive foreground, actors are interested in satisfying basic individual needs. The *normative* goal frame, in contrast, facilitates the generation of collective goods and implies sensitivity toward social expectations, reputation, and reciprocity. The mindreading, or “mentalizing” (Gamble et al. 2014:161), virtuosity “to put oneself in the shoes of others” is perhaps one of the most important mental changes in human evolution (Lindenberg 2015b:6). Its signature “is enhanced by the fact that it is linked to social emotions, such as guilt, shame, and gratitude” (Lindenberg 2015a:50). Finally, the *gain* goal frame is a mindset related to investments into one’s future resources, made possible “by the ability to put oneself into the shoes of one’s own future self” (Lindenberg 2015a:50). Since the gain goal frame is weaker than the hedonic goal frame, it needs institutional support, as Max Weber (1972) highlighted in his famous elaboration of the modern, Western conduct of life (Lindenberg 2013:85). These three overarching goal frames are often antagonistic, and their calibration requires considerable self-regulatory capacities. Each goal advances to the cognitive foreground by pushing the other goals to the cognitive background, whereby the relative importance of each goal depends on the perceived situational context (Fig. 2).

Lindenberg’s goal-framing theory does not simply assume an internalization of norms. It depends on situational cues as to whether a person is in a normative goal frame or not. If he or she *is* in a normative goal frame, the subsequent problem is to find out which behavior is required in order to meet others’ expectations. To this end, a mental model represents the normative aspects of the situation (Lindenberg 2008). However, the stability of normative goal frames requires compatible gain-related or hedonic goals in the background (Lindenberg 2008:70)—in other words, the desire to avoid public shaming. Goal-framing theory is focused on actors’ capacities to calibrate these goals in a situationally appropriate way, rather than distinguishing between slow, automatic-unconscious vs. fast, deliberate-conscious cognitions (Kahneman 2012). It fits better to the analysis of social order and ties in social exchange networks because there is usually a considerable timespan between gift and return (e.g., birthday party invitations; see below). The *normative goal frame* is active when actors discuss or think about situations where reciprocity is expected. Humans are social creatures. They tend toward “hot cognitions” (emotions) in social situations such as joint laughter. In “hot cognitions,” neuropeptides (Gamble et al. 2014:58) provide a neuropharmacological basis for prosocial behavior. Their effect, however, is *parochial altruism* (Bowles and Gintis 2011) rather than unlimited prosociality—neuropeptides make us more prosocial within our own group, but potentially more aggressive toward out-group members (Sapolsky 2018:116). Since group-mindedness and “strong solidarity” mostly apply to the in-group, perceived group boundaries possibly moderate our disposition to reciprocate, also because of the influence from background goals (Lindenberg 2013:84), which can weaken the normative goal frame.

Within societies, there usually is sufficient taken-for-granted consensus about which goal frame should be in the cognitive foreground in a given social situation. This consensus might differ, however, between cultures and societies: “It does happen—particularly in complex, multiethnic, rapidly changing societies—that two or more people interacting with each other are applying different models to any given aspect of the interaction. When they do this, recriminations, conflict, and a breakdown of trust almost inevitably result, because adherence to one model usually violates the standards of any other” (Fiske 1992:712). For instance, one party regards an interaction in terms of authority ranking (e.g., with respect to gender roles), whereas the other party applies the normative goal frame of reciprocity. Goal-framing theory suggests that such situations can negatively affect the higher-order self-regulation concerning the clarity of one’s own self-schema. Cultural confusion can reduce the clarity of a person’s cultural self—that is, who one actually is—which can have negative consequences for the capacity to calibrate goal frames (Lindenberg 2013:96). On the other hand, our evolutionary past as hunters and gatherers favored cognitive conditions for “fission-fusion” processes, particularly parochial altruism, but at the same time a remarkable flexibility of group identification (Lindenberg 2015c:34, 41). When societies became larger, more inclusive, and more diverse, exchange between different people and groups required “weak solidarity,” meaning that the “legitimately expected sacrifice for solidarity is likely to decline with increasing inclusiveness” (Lindenberg 2015c:48). Although we are social beings, our inclination toward solidarity declines the more different—culturally or socially—the respective other is. Humans do not just interact in small groups, as apes do. We are, in J. Haidt’s (2012:220) words, 90% chimp and 10% bee. The 10% bee component enables us to go beyond our 150 familiar network ties (Dunbar 2003:172) and to interact in highly inclusive, anonymous, and culturally diverse mass societies (Turner 2021:243), for which we *need* “weak solidarity” and a flexible adjustment of our goal frames. From this perspective, ethnic network segregation results from different ways of how persons or groups calibrate their mindsets and how they construct mental models of the normative aspects of the situation (Lindenberg 2008) during an episode of exchange.

Network segregation along ethnic lines partially results from selective *choice*, given the opportunity structure, limited information, and perhaps a higher risk of signal error in interethnic dyads (Sapolsky 2018:348) during the initiation of a relationship. Moreover, if the normative goal frame is weaker in interethnic dyads and the normative aspects of the situation have a different meaning than in intraethnic dyads, the likelihood of reciprocity might also be lower than in same-ethnic dyads (Fig. 2).

When deciding on reciprocity, the normative goal frame is active at each moment—for example, when parents and children discuss whether a particular child should be invited to a birthday party or not. On the one hand, the normative goal frame is difficult to override or to push back into the cognitive background (Lindenberg 2015a) in social situations. On the other hand, “weak solidarity” in highly inclusive and ethnically diverse societies makes a difference between intra and interethnic social exchange, which might further stimulate ethnic network segregation and thereby further rigidify ethnic boundaries.

The concept of ethnic boundaries explicitly disagrees with essentialist views of ethnic groups and cultures, instead highlighting the social-constructivist aspects of intergroup relations (Barth 1969; Wimmer 2013). Ethnic boundaries are subject to agency, to practices, identity politics, and response to stigmatization (Lamont and Mizrachi 2012) but can also result in “assimilation” when these practices blur group boundaries in the long run (Alba and Nee 1997; Verkuyten 2013:67). If the commitment to reciprocity strives for the cognitive foreground and starts framing the situation (Lindenberg 2013), substantial cognitive and emotional effort will be required to deny a reciprocation (Rilling and Sanfey 2011) once the actor has accepted a gift.

According to “parochial altruism” (Bowles and Gintis 2011) and “weak solidarity” (Lindenberg 2015a), the ethnic category of the other person might moderate our social preference for reciprocity. Once an episode has started, the normative goal frame (Lindenberg 2013) and “neurologically wired” social cognition (Greene 2015: 45) become important drivers of ties in intra *and* interethnic dyads, but this cognition makes a difference between in-group and out-group interaction. Network segregation along ethnic boundaries results from two mechanisms: first, from (limited) rational choice when persons bring an exchange episode into operation, given the uncertainty in intercultural settings (Fiske 1992; Sapolsky 2018:348), opportunities, and third-party intervention (Windzio 2018). Second, ethnic network segregation results from a *combined* effect of reciprocity and parochial altruism during an ongoing episode if the normative goal frame is not sufficiently supported by the background goals in interethnic dyads, or if the normative aspects of the situation are not interpreted in a way that triggers reciprocity.

In a study analyzing longitudinal networks of children’s visits at their peers’ homes, a continuous-time simulation of a stochastic actor-oriented model (SAOM) showed a strong effect of *reciprocity on tie creation* (Windzio 2018). In the empirical part of the present study, in contrast, social networks of adolescents’ birthday party invitations will be analyzed, where children and gifts are exchanged and the norm of reciprocity is generally high. Once a person has accepted an initial offer, however, he or she becomes liable to the strong norm of reciprocity. The normative goal advances to the cognitive foreground (Lindenberg 2013) and the disposition toward reciprocity becomes important in the process of tie formation. Considerable cognitive effort is now required to override the normative goal.

In the longitudinal analysis below, the effect of reciprocity will be tested not only for intraethnic dyads, but also for dyads of any other ethnic constellation. Combining the normative goal frame of strong reciprocity with parochial altruism and “weak solidarity” leads to the hypothesis that the reciprocity effect is considerably smaller in *inter*ethnic than in *intra*ethnic dyads.

## Data and Methods

### Longitudinal School Class–Based Network Data

The empirical analysis is based on longitudinal, school class–based network panel data. The data has been collected for grades 5, 6, and 7 in the years 2011, 2012 and 2013 in the German city-state of Bremen. Pupils’ average age over this period is 11.5 years. The focus of this study is the interplay of multiplex networks (e.g., friendship, birthday parties, or ties among parents), ethnic segregation, educational achievement, and well-being in school. Response rates of pupils varied from 75.4% in wave 1 to 80.4% in wave 3. Since the participation of schools depended on the school principals’ consent, as well as on the teachers’ willingness to support this study, there was considerable nonresponse at both the school and class level; one third of all classes in the population did not participate. Since the quality of social network data depends on participation rates within classes, only classes in which either 75% or at least 17 pupils participated have been analyzed. The final analysis was limited to classes that participated in all three waves, so a maximum of 501 students in 21 school classes were available for the analysis.

Pupils completed the questionnaire under the guidance of the interviewer in the classroom, so information on networks is available within classes. In accordance with data privacy regulations, the network generator worked in the following way: Clearly visible ID numbers were placed on each desk and lists with first names and numbers were stored in the schools in order to link the observations between the panel waves. Reliability analysis supports the procedure: Matching the information on ego’s attendance at alter’s birthday party from both perspectives—hosts and guest—leads to a rate of agreement of 95.44% (wave 1), and a good interrater reliability of 0.725 (Cohen’s kappa coefficient). Tables 1 and 2 show the descriptive statistics of the dyadic and the actor attributes used in the empirical analysis.

**Table 1:** Descriptive statistics

Variable	Item	Coding	density, $\overline{x}$	SD	min	max
Birthday party	“Whose birthday party did you attend?”					
(W1)		network	0.078	–	0	1
(W2)		network	0.141	–	0	1
(W3)		network	0.169	–	0	1
Ego lives close to alter (5 min.)	“Who lives near you so that you can walk there in 5 min or less?”					
(W1)		network	0.049	–	0	1
(W2)		network	0.065	–	0	1
(W3)		network	0.072	–	0	1
Ego nominates alter as friend	“Who are your friends?”					
(W1)		network	0.232	–	0	1
(W2)		network	0.268	–	0	1
(W3)		network	0.259	–	0	1
Contact among parents	“Do your parents know the parents of other students in your class?”					
(W1)		network	0.054	–	0	1
(W2)		network	0.045	–	0	1
(W3)		network	0.042	–	0	1
Girl	“Are you male or female?”	1 = yes, 0 = no	.47	–	0	1
Grade-point average	“Enter your school grades from your last certificate for the … subjects” (Math, German, English)	5 = very good; 0 = insufficient	2.93	.80	0	5

*N* (individuals) = 501, *k* (classes) = 21, *t* (waves) = 3

**Table 2:** Descriptive statistics, ethnicity

	*N*	%	Cumulative %
Germany	398	79.44	79.44
Turkey	28	5.59	85.03
Poland	7	1.4	86.43
Serbia/Croatia/Bosnia	4	0.8	87.23
Russia/Kazakhstan/Ukraine	19	3.79	91.02
Africa	6	1.2	92.22
Other	39	7.78	100

*N* (individuals) = 501, *k* (classes) = 21, *t* (waves) = 3

### Modeling Network Evolution

Stochastic actor-oriented models (SAOM) have been developed for the empirical analysis of network evolution over time (Snijders et al. 2010). Since panel data is discrete in time (see Fig. 1), SAOMs estimate the parameters by simulating microsteps between discrete measurements. Based on the model specification and the empirical data, the SAOM simulates actors’ decisions during a microstep. They can decide to dissolve or establish a link, or maintain the presence or absence of a tie in a multinomial logit choice model. The model estimates effects on log odds of creation, dissolution, and maintenance of ties. In line with the assumption that each “actor has his or her own goals which he/she tries to advance in accordance to his/her constraints and possibilities” (Snijders 1996:149), actors’ decisions depend on the evaluation of the utility of each option. Starting values for the utility function are taken from the first observation of the network at time *t* and become updated with the empirical information of the network at time *t* + 1 … *t* + *k*. For instance, if the first network shows a higher tendency toward reciprocity or gender homophily, a high utility weight will be assigned to decisions in favor of these states. Models run separately for each network, so results are combined in a random effects (RE) meta-analysis. Owing to nonconvergence in some networks, the final meta-analysis is based on a maximum of 18 networks.

**Fig. 3:**
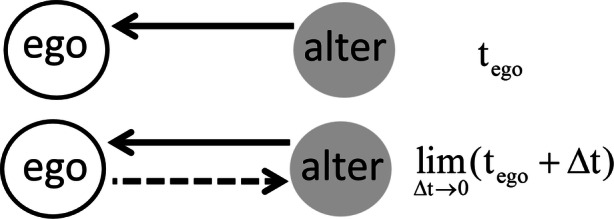
Effect of reciprocity on tie creation in a microstep at a given simulated moment

Fig. 3 illustrates the effect of reciprocity on tie creation in the SAOM. During a microstep in a given moment *t*_ego_ during the simulation, the focal actor “ego” already has an incoming tie from alter. If the utility of reciprocity on tie creation is high, ego will be highly inclined to reciprocate at *t*_ego_ + Δ*t* (dashed line in Fig. 3), given that there is no better alternative, according to the utility function. Keep in mind that the term “utility” in the theory of the SAOM comes from the econometric literature on discrete choice-models and does not mean that people always behave in a perfectly “rational” way (Greene 2008:842). It has been argued in the present study that reciprocity results from the normative goal frame, which also has a “wired” emotional component. However, since “weak solidarity” and more-abstract norms gained in relevance in modern and diverse societies (Lindenberg 2008), there is increasing variation in how people construct mental models of the normative aspects of situations. Not to reciprocate is costly with regard to reputation and feelings of shame, but possibly less costly in interethnic dyads given the tendency toward parochial altruism and weak solidarity. This effect can be identified by estimating the interaction term “reciprocity on creation × same ethnicity.”

## Results

Table 3 shows four SAOM meta-analyses of the evolution of birthday party networks. Despite the simplicity of the SAOM model specification, the models and data fit quite well, which is also due to strong and robust effects of the dyadic covariates “spatial proximity” and “friendship ties” (that is, other *networks* as explanatory variables). These networks capture an important part of network structural effects. Models 1 and 2 estimate the effect of “reciprocity” on creating a tie; models 3 and 4 estimate its effect on maintenance of a tie. Each first model (M1 and M3) estimates only the main effect of reciprocity on tie creation; each second model (M2 and M4) estimates the interaction effect “reciprocity × same ethnicity.”

**Table 3:** Log odds of ties in birthday party networks (ego to alter). SAOMs, grades 5, 6, and 7. Random effects meta-analysis

	*reciprocity: creation*		*reciprocity: maintenance*	
	M 1 *k* = 18	M 2 *k* = 15	M 3 *k* = 17	M 4 *k* = 7
outdegree (density)	−2.8139**	−2.7223**	−2.9735**	−2.9407**
reciprocity (*creation*)	1.7439**	1.1325**	–	–
reciprocity (*maintenance*)	–	–	−0.0018n.s.	0.6252n.s.
transitive triplets	0.311**	0.3263**	0.4078**	0.3159**
*Dyadic effects*				
friendship ties	0.7196**	0.7270**	0.8531**	0.8643**
spatial proximity (5 min. walk)	0.2359+	0.2656*	0.2770+	0.3857n.s.
contact among parents	0.3812*	0.3393+	0.4911**	0.3718+
*Similarity effects*				
both are girls	0.8671**	0.8769**	0.9739**	1.1077**
same ethnic origin	0.2300**	0.1121n.s.	0.3054**	0.2777+
grade similarity	0.1572n.s.	0.1336n.s.	0.1725n.s.	0.0848n.s.
*Interaction effects*				
reciprocity × same ethnic origin *(creation*)	–	1.0365**	–	–
reciprocity × same ethnic origin *(maintenance*)	–	–	–	−0.9718n.s.

+ *p* ≤ 0.1; * *p* ≤ 0.05; ** *p* ≤ 0.01; *** *p* ≤ 0.001

**Fig. 4:**
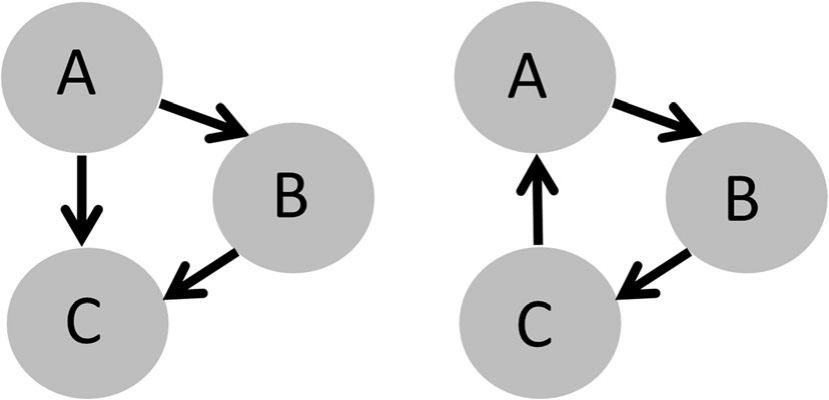
Transitive and cyclic triplets

To begin with, we focus on the effect of reciprocity on tie creation in M1 and M2. First and foremost, both models show a strong, positive and highly significant effect of reciprocity on tie creation. In addition, the effect of transitive triplets has been estimated. Transitive triplets are transitive triads in which there is a tie from actor A to actor B, from actor B to C, and from actor A to C (Fig. 4). In contrast to cyclic triplets, with ties from A to B, from B to C, and from C to A, transitive triplets are a main structural characteristic of human social networks. They are a network-structural expression of social-cognitive balance in terms of “friends of my friends are my friends” (Prell 2012:143; Windzio 2015). Transitive triplets show a positive and highly significant effect. The same is true for spatial proximity (5 min walking distance, or less) between ego’s and alter’s residential locations and the effect of friendship ties on birthday party networks. Unsurprisingly, the latter is strong, significant and positive as well.

Birthday party networks are segregated along gender lines. The effect of “both are girls” is strong and significantly positive in all four models: the log odds of observing a tie in the birthday party network are considerably higher for two girls compared with dyads of two boys, or mixed dyads. Overall, we also find a positive effect of similarity in grades in M1, but the effect turns insignificant in M2. Furthermore, spatial proximity and contact among parents tend to increase the log odds of a tie in the birthday party network.

Important for our research question is the interaction effect in M2. Here, the interaction of ethnic categories with reciprocity on tie creation is significant and positive. How should we interpret this interaction? In M2, the odds ratio (OR) for a particular covariate constellation results from combining both terms—main effects *and* interaction effect.

$$ {\displaystyle \begin{array}{c}\mathrm{same}\ \mathrm{ethnicity}=\exp \left(1.1325+0.1121+1.0365\right)=9.78\\ {}\mathrm{other}=\exp (1.1325)=3.10\end{array}} $$

**Fig. 5:**
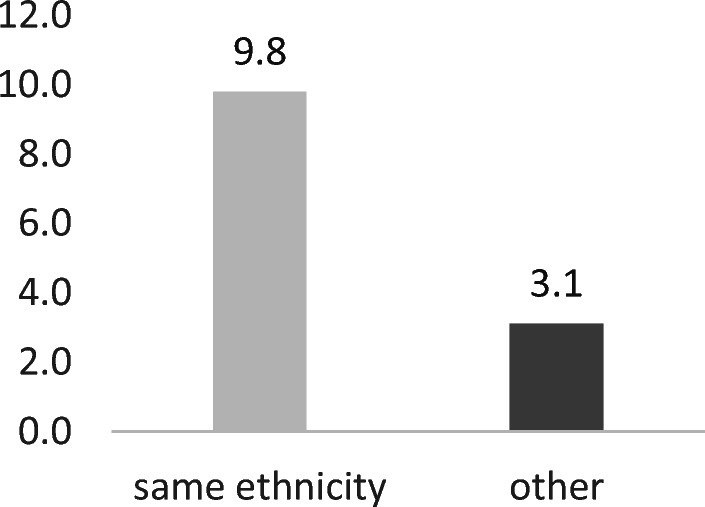
Odds ratios of reciprocity on tie creation by “same ethnicity” (M2)

Figure 5 gives a visualization of the overall effect. Recall that the effect of reciprocity on tie creation is the effect of an incoming tie on reciprocation when it has not yet been reciprocated (Fig. 3). It is the reciprocity effect compared with a situation without an incoming tie. The overall effect of reciprocity on tie creation is very strong in dyads of same ethnicity (OR = 9.78), but reciprocity is also strong in all other dyadic ethnic constellations (OR = 3.10). Following from this, boundary-crossing social exchange is certainly possible.

Actors deliberately decide whether to start an exchange episode or not, and whether to offer or accept a “gift” (Mauss 1967) or not. Given their preferences, they evaluate the utility of becoming involved and develop a subjective expectation of how likely they are to realize benefits by doing so (Windzio 2018). Once an exchange episode has come into operation, in contrast, the strong tendency toward reciprocity drives the further evolution of the network. According to the theoretical arguments, network self-organization by reciprocity and the normative goal frame become important during the exchange process. Overriding the disposition toward reciprocity, which is ingrained in human biology (Rilling and Sanfey 2011:30), requires cognitive and emotional effort.

This tendency toward reciprocity is generally strong, also in interethnic dyads, but *particularly* strong in intraethnic dyads. An additional mechanism of network segregation along ethnic boundaries is thus the ongoing exchange process and the difference in the tendency toward reciprocity between intra- and interethnic dyads, which might result from “weak solidarity” in more diverse and inclusive societies (Lindenberg 2015c).

In models 3 and 4 in Table 3, “maintenance” means that a tie which is already mutual will be kept at a given moment. This is not the case empirically. The results highlight the importance of a strong commitment to reciprocity, but having a mutual tie does not necessarily result in a sustainable social relationship in the birthday party network.

Ethnic origin can affect network segregation when making or accepting a gift during the initiation of a tie, but also when deciding on reciprocity during the ongoing exchange. Goal-framing theory makes an important contribution to reconceptualizing network ties in a longitudinal perspective. Of course, in our daily activities several cognitive modes are always active: the hedonic, gain, and normative goal frames (Lindenberg 2015a). During an ongoing exchange episode, actors sometimes think about whether or how they should further engage. Network self-organization at the macro level results from the fact that the relevance of a cognitive mode can change in the course of an episode of social exchange at the micro level—or, in other words, the shifting of a goal frame between cognitive foreground and background. If the normative goal frame is in the cognitive foreground, actors must invest considerable emotional and cognitive effort to “override” the biologically ingrained tendency to reciprocate (Rilling and Sanfey 2011), once an initial offer has been made. On the other hand, the normative goal frame becomes weaker in interethnic dyads in highly diverse and inclusive societies. Interestingly, results in Table 3 do not show any significant effect of reciprocity on the maintenance of ties. Consequently, the strong tendency toward reciprocity exists only in each particular round of “tit-for-tat”—cards seem to become reshuffled after reciprocation of an invitation.

## Summary and Conclusion

Recent developments in social network analysis focus on the evolution of networks over time. Longitudinal network data in combination with appropriate simulation methods of network evolution between discrete measurements leads to a redefinition of a core concept in social network analysis, namely the *edge* or *network tie*, the link between two nodes. Focusing on exchange relationships gives way to the analysis of social network ties as *episodes* (Molm et al. 2012). Episodes have a starting point, they proceed over time, and they usually also have an ending point. Having dissected a social exchange episode into different elements, we can identify different mechanisms of how network segregation along ethnic boundaries emerge.

Following the arguments in the theoretical section of this study, *rational choice* might explain the initiation of a social exchange episode, given the respective opportunities, whereas the normative goal frame of *reciprocity* is important during the ongoing episode. In social network analysis, the term “self-organization” describes the macrolevel manifestation of our ingrained tendency toward reciprocity at the microlevel. Based on neurophysiological structures, reciprocity in networks is definitely an important aspect of a network’s self-organization. Reciprocity is also a strong and robust effect in birthday party networks. It has been estimated on the *creation* of ties, which indicates the propensity to reciprocate a tie when it has not yet been reciprocated. The effect of reciprocity is generally strong, but even stronger in dyads of same ethnicity (OR: 9.78 vs. 3.10).

The interaction “reciprocity × creation” with same ethnicity partly explains ethnic segregation in networks: it represents the process of reciprocity within one’s own group, whereas the reference group “other” represents the dynamics of *boundary-crossing* social exchange. These differences in reciprocity on tie creation within and between ethnic groups show ethnic boundaries at work.

First, network segregation along ethnic boundaries results from the selection of partners with whom actors *initiate* a social exchange relationship. This is usually driven by the hedonic or gain goal frame (Lindenberg 2015a), given their respective opportunity structure. The initiation period of the exchange episode ends when the initial offer (e.g., an invitation to a birthday party) has been accepted. Second, accepting an initial offer activates the normative goal frame. According to the considerable strength of the reciprocity effect on tie creation, even in the ethnically heterogeneous reference group “other” (OR: 3.10), it seems to be difficult not to reciprocate once an initial offer has been accepted. Nevertheless, following from the difference between these effects, an important part of the ethnic segregation of networks is due to the fact that boundary-crossing exchange has a lower likelihood of being reciprocated. It is known from cooperation experiments that actors “favor in-group members not because of altruistic sentiments toward those who are similar to themselves, but because they expected reciprocation from in-groupers and not from out-groupers” (Bowles and Gintis 2011:36). This is in accordance with Greene’s argument that less positive emotions and negative associations are more prevalent in intergroup relations (Greene 2015:54, 69), even though, of course, “being wired for tribalism does not mean being *hardwired* for tribalism” (Greene 2015:55). Calibrating different goal frames depends on the capacity to self-regulate. Self-regulation is a crucial aspect of Dunbar’s “social brain” (2003), which, in turn, is a product of human evolution (Boehm 2012). Social networks and ethnic network segregation are indeed results of our social brains.

The result is also in line with the distinction between “leap of faith” and “testing the water” (Kuwabara and Sheldon 2012)—interethnic social exchange seems to be closer to the latter type. Regarding network ties dynamically, as exchange episodes, is appropriate to an agency theory based on dynamic cognition: hedonic or gain goal frames are in the cognitive foreground when deciding to start an exchange episode or not, given the opportunity structure, whereas norm-oriented behavior moves to the cognitive foreground when responding to an initial offer and deciding on reciprocation. In modern, inclusive and diverse societies, “weak” solidarity leaves uncertainty on normative aspects of social situations. Therefore, hedonic and gain goal frames sometimes remain strong in the cognitive background. Proponents of rational choice could object that neuronal activity remains unobserved in network studies based on survey data. Fair enough, but the same applies to actors’ internal computations (e.g., in prisoners’ dilemmas). Contrariwise, given the evidence cited in this study, it is rather difficult to explain why the neural basis of different cognitive modes should *not* influence ethnic boundaries.

Combining social network analysis, goal-framing theory, and research on ethnic boundaries is not yet common in the field of immigrant integration research. Hopefully, an interdisciplinary perspective is currently emerging (Lamont et al. 2017). Future research should consider more systematically the contextual embeddedness of network processes, and studies on ethnic boundaries should combine the episode concept of network ties with network ecologies, as it has been done in a recent study (McFarland et al. 2014).
